# Levels of cooperation increase during interactions with iterated decision-making

**DOI:** 10.1093/beheco/arag121

**Published:** 2026-09-30

**Authors:** Letizia Pessina, Rufus A Johnstone, Mélisande Aellen, Redouan Bshary

**Affiliations:** Faculté des Sciences, University of Neuchâtel, Emile-Argand 11, Neuchâtel 2000, Suisse; Department of Zoology, University of Cambridge, Downing St, Cambridge CB2 3EJ, United Kingdom; Brain and Cognitive Sciences Department, University of Rochester, 500 Wilson Blvd, Rochester, NY 14611, United States; Faculté des Sciences, University of Neuchâtel, Emile-Argand 11, Neuchâtel 2000, Suisse

**Keywords:** cooperation, mutualism, *Labroides dimidiatus*, *Labroides bicolor*, decision-making, tolerance, iterated prisoner's dilemma

## Abstract

Models on the evolution of cooperation typically assume that each interaction consists of a single decision. However, many real-life examples of intraspecific cooperation and interspecific mutualism consist of prolonged interactions in which partners make multiple decisions. This begs the question of how levels of cooperation change within a single interaction over its course. We model and empirically explore a case in which the game structure is asymmetric: a service provider (a cleaner fish) takes multiple foraging decisions on a “client” reef fish. If the cleaner eats a parasite it cooperates, if cleaner eats a bit of client mucus it cheats. Clients, in turn, may decide to stay or to terminate the interaction but have no means to cheat the cleaner. Despite the fact that parasites become less and less available, the model predicts that cleaners refrain more from mucus eating as an interaction progresses. This is an adaptive response to the clients' increased readiness to terminate the interaction as their parasite load decreases. Both field observations and laboratory experiments on cleaner wrasse *Labroides dimidiatus* and *L. bicolor* confirm the model's prediction. The results in this game with asymmetric cheating opportunities are opposite to human behavior in symmetrical iterated prisoner's dilemma games where players cooperate less when the end is near.

## Teaser

We studied cleaner fish that repeatedly choose whether to remove parasites or bite client mucus. Most theories assume cooperative interactions involve a single choice, yet as in this case many real interactions involve repeated decisions. Our model and experiments show cleaners become more cooperative over time because clients are increasingly likely to leave once their parasite load drops. These fish thus cooperate more as interactions progress.

## Introduction

Cooperation between unrelated individuals, including from different species, often involves investments, ie behaviors that reduce the immediate payoff of the actor and benefit others (Bergmüller et al. 2007; West et al. 2007; Bshary and Bergmüller 2008). Such investments can only be under positive selection if they yield future benefits to the actor (Bergmüller et al. 2007; Bshary and Bergmüller 2008; Raihani and Bshary 2011). To explain how these future benefits are secured, classical game theoretic models, such as the Prisoner's dilemma, typically model an encounter between partners as an interaction consisting of a single, discrete decision (eg, to cooperate or to defect). Because a single decision offers no chance to recoup an investment, these analyses emphasize the need for iteration, repeated interactions with the same partner over time, in order to have conditionally cooperative strategies as potential evolutionarily stable strategy (ESS) (Axelrod and Hamilton 1981; Axelrod and Dion 1988). Indeed, the possibility of future encounters with the same partner, known as “the shadow of the future”, should support cooperation (Bó 2005). Conversely, when there is no future interaction to be expected, actors should switch to defection (Bó 2005). In line with this logic, many economic experiments on humans report such “end game effects”, ie cooperation gradually deteriorates in games with finite horizons (Milinski et al. 2002; Bó 2005; Normann and Wallace 2012).

However, while stylized game theoretic models assume that each interaction consists of a single decision, this overlooks a crucial biological reality: many real-life social encounters are prolonged events in which individuals make multiple, sequential decisions within a single, continuous interaction. This means that individuals can adjust their own behavior to that of partners in real time. For example, lionesses may slow down their approach to an intruding neighbor pride if their own partners lag behind (Heinsohn and Packer 1995). Chimpanzee males will adjust their own actions during a hunt on monkeys according to what other males do, as well as how monkeys respond (Boesch and Boesch-Achermann 2000). Another case study involves cleaner fish of the genus *Labroides* and their client reef fish (Bshary and Cote 2008). During an interaction, cleaners repeatedly eat items off the surface of clients. Cleaners make a cooperative act when they remove an ectoparasite, and they make a defective act when they take a bite of protective client mucus (Ebran et al. 1999; Reverter et al. 2018; Bshary and Noë 2023). Eating mucus is indicated by a jolt of the client's body (Bshary and Grutter 2002). Both parasites and mucus can be found in the stomachs of cleaners, showing that cleaners eat both food types on a regular basis (Randall 1958; Grutter 2001; Oates et al. 2012). Clients may respond to cleaner foraging decisions by terminating an interaction through aggressive chasing of the cleaner or by swimming off (Bshary and Grutter 2002). These examples raise the question of how levels of cooperative behavior might change during an interaction, and whether or not one should expect end effects as well, potentially depending on whether future interactions are likely to occur. Here, we model this problem and provide empirical data. The stability of the cleaning mutualism has been modeled before (Johnstone and Bshary 2002, 2008). Due to repeated decision-making within a single interaction, mutually beneficial outcomes can be achieved in principle even in a so-called one-shot game (Johnstone and Bshary 2002), ie without the need for repeated interactions. The precise outcome depends on how much control clients have to terminate the interaction against the will of cleaners, and to what extent the value of a bite of mucus is higher than the value of an ectoparasite (Johnstone and Bshary 2002). The models can explain why mutualistic cleaning interactions occur even when clients are pelagic or when cleaners rove over large areas, ie when future interactions are unlikely or at least cannot be planned (Johnstone and Bshary 2002). Within the genus *Labroides*, adult *L. bicolor* are roving cleaners, while adult *L. dimidiatus* have defined territories, termed “cleaning stations” (Randall 1958; Potts 1973). What the models do not predict is how levels of cleaners' cooperation change over the course of an interaction, and whether the general tendency (increasing or decreasing levels) depend on whether repeated interactions can be planned or actively avoided.

For our current model, we assume that clients may terminate interactions after each cleaner foraging act by swimming off. Swimming off is a behavior that visitor client species with access to several cleaning stations typically do in response to cleaners taking a bite of mucus (Bshary and Grutter 2002). We do not model how the response of resident clients with access to a single cleaning station to mucus feeding, which consists typically of chasing the cleaner (Bshary and Grutter 2002), may alter cleaner fish decisions. Regarding predictions of what should happen, two relevant forces may cause opposite predictions. First, basic optimal foraging theory would view clients as a food patch that gets depleted over time during an inspection (Charnov 1976). Given that cooperative behavior, ie parasite removal, is indeed a proximate cause for which clients seek out cleaners (Grutter 2001), increasing parasite depletion would decrease the initial high quality of a client leading to an increase in the temptation for a cleaner to cheat and take a mucus bite (Johnstone and Bshary 2002; Triki et al. 2022). Thus, cheating rates should increase during an interaction. Second, one has to consider clients as active players in interactions. They can respond to cleaner's actions mostly by deciding to stay or to leave (Bshary and Würth 2001; Bshary and Grutter 2002, 2005). Their decision rules could hence determine how cleaner fish should behave (Johnstone and Bshary 2008). The value a client gives to the continuation of an interaction will depend on its parasite load: the higher the load, the more the client can gain from continuing the interaction. Therefore, the value is highest at the beginning of an interaction, and it decreases as the cleaner removes ectoparasites. As a consequence, clients should be more tolerant towards a cheating act by a cleaner early in an interaction, and less so later on. Cleaner fish, on the other hand, may have expectations about a client's parasite load based on past experience with members of the same species or size but do not know the precise number nor the location of each parasite, which introduces further variation in foraging efficiency. However, if cleaners generally respond to client responsiveness, they should become more cooperative as interaction time increases. Indeed, clients are more tolerant towards a cheating cleaner if highly parasitized (Bshary and Grutter 2002), a tendency which was recently modeled by Triki et al. (2022).

In addition to the temptation for cleaners to cheat and client tolerance towards acts of cheating, the probability that current interactions affect the likelihood of future interactions might play a role. In territorial *L. dimidiatus* (Randall 1958; Potts 1973; Grutter 2001), a cleaner cheating a visitor client may not only cause the termination of the interaction but in addition cause the victim to visit a different cleaner for the next inspection leading to the loss of a future client (Bshary and Grutter 2002, 2005). The larger the “shadow of the future”, the more important it might be to avoid a late cheating act causing important harm in the future. Indeed, observations showed that clients are more likely to return if the previous interaction ended without conflict (Bshary and Schäffer 2002; Soares et al. 2013). As adult *L. bicolor* roves over large territories (Randall 1958; Oates et al. 2010a, 2012), clients cannot readily apply partner choice as a function of service quality (Mills and Côté 2010). Clients might in fact not be able to find the same cleaner again for their next inspection, or might encounter the cleaner they try to avoid at a different location. Roving behavior also means that *L. bicolor* more actively approaches clients rather than waiting for them to visit, giving it more power to enforce interactions (Oates et al. 2010a, 2012). For all these reasons, the “shadow of the future” appears to be of comparatively minor importance for any decisions about current service quality in *L. bicolor*. As a consequence, one might expect more “end game effects” like in human studies in *L. bicolor* than in *L. dimidiatus* (Milinski et al. 2002; Bó 2005; Normann and Wallace 2012).

To complement the model, we conducted both field observations and an experimental study in the laboratory with the two cleaner species. For the observational study, the two cleaners were observed interacting with their clients in the wild. We identified interactions in which at least two cheating events occurred, and compared how long it took the cleaner to cause jolts as correlates of cheating the first and the second time. In addition to such correlative data, we conducted an experiment in the laboratory in which cleaners were offered discreet items of preferred prawn and less preferred flakes. Cleaners had to eat against their preference in order to prolong interactions. We quantified the number of flake items eaten before the first prawn item, and after the first until the second prawn item, to evaluate how the cleaners' willingness to eat against preference changed during the course of an interaction.

## Materials and methods

### The model

The model focuses on a single interaction between two individuals, only one of which is a potential exploiter (the cleaner), while the other is a potential victim (the client). Exploitation imposes an additive fitness cost on the victim, while conferring an additive fitness benefit on the perpetrator. The payoff to both from their encounter depends upon the level of exploitation (biting of live tissue) by the former, which may vary over the course of the interaction. We will write *x* (*t*) for the level of exploitation at time *t*. A basic assumption of the model is that a client benefits from cooperative interactions with a cleaner, but that the marginal rate of gain decreases over time, eventually hitting zero, which is the latest point at which the client will depart, ie even if the cleaner is not engaging in any exploitative behavior. For simplicity, we assume a linear decrease in marginal benefits. Formally, client marginal benefit is denoted *b*(*k*, *t*), where *k* is a parameter that specifies client “need”, and we will assume for simplicity that *b*(*k*, *t*)=Max{*k* − *t*, 0}. Another model assumption is that clients will vary in their parasite load and hence need for cleaning. We typically assume that such variation is captured by a Beta distribution but will also present a case where a lognormal distribution is assumed. A client's current need is captured by the parameter *k*, which determines the initial marginal benefit of interaction with the cleaner, and hence the length of maximal interaction time until the client would choose to depart. In the analyses, *k* ranges from 0 to 1, and follows a Beta distribution. Only the client knows its current need, the cleaner does not. This assumption is reflected in the fact that ectoparasites are rather cryptic and must be searched for. While cleaners may have mean expectations based on client body size and/or species (Gorlick 1984; Grutter 1995, 1996; Arnal et al. 2001; Eckes et al. 2015; Roche et al. 2021), this aspect is not a focus for our current research questions. Figure 1 summarizes model assumptions regarding the client perspective.

**Figure 1:**
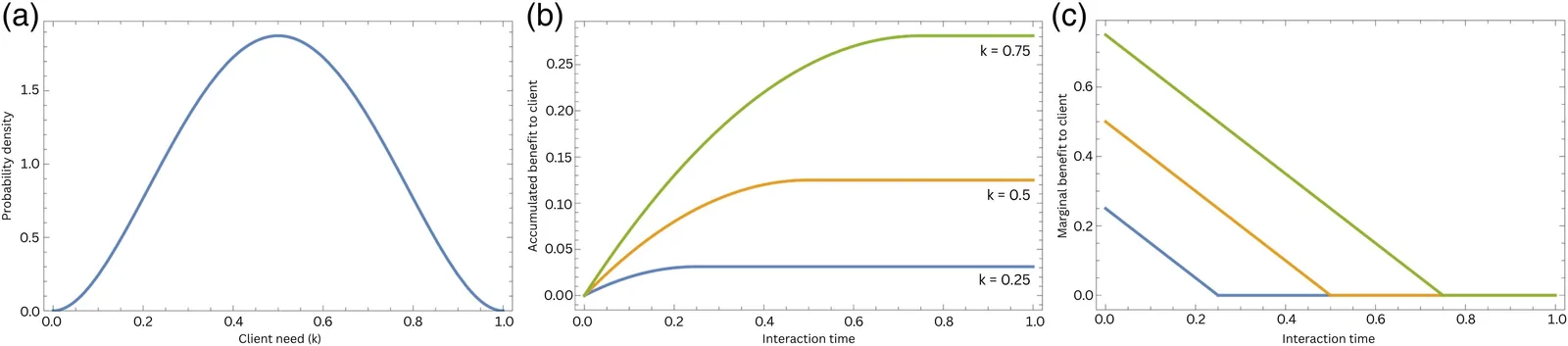
Model assumptions regarding the clients’ need to receive cleaning. a) The Beta distribution of client need *k*, which is a function of their parasite load; b) diminishing return functions for different values of *k* as a function of interaction duration; c) the marginal benefits to clients as interactions proceed depending on different values for *k*. The values of *k* reflect either low (0.25), moderate (0.5) or high (0.75) need for cleaning.

For a cleaner, we assume that it can choose to exploit the client, at a varying rate of intensity. For simplicity, we ignore the benefits that arise from eating ectoparasites and suppose that the cleaner's payoff is just the sum total of exploitation over the course of the interaction (up until the point where the client leaves). Thus, cleaners do not derive any “cooperative” benefits from interacting with clients, they just benefit from exploitation. Exploitation imposes an additive cost on the client, so that its net marginal benefit from continued interaction is reduced, and hits zero earlier, causing the client to leave sooner. Thus, the cleaner has to balance the greater rate of gain from more exploitative behavior against the reduction in interaction time it will cause. We model this as the exploitation intensity, ie the probability that a cleaner will take a mucus bite instead of removing an ectoparasite. This probability can in principle vary between 0 and 1. Our key question is how cleaners modulate exploitation intensity over the course of an interaction.

The marginal cost to the victim of prolonging the interaction beyond time *t*, as a result of exploitation, is equal to *x* (*t*) (the level of exploitation at that time). Consequently, the net marginal benefit obtained by the client at time *t* (taking into account the effects of exploitation by the cleaner), denoted *w*(*k*, *t*), is given by *w*[*k*_, *t*_] = *b*[*k*, *t*] − *x*[*t*].

The model we present does not include a parameter that would capture the likelihood that cleaner and client meet again and remember each other's behavior during past interactions. Thus, we did not incorporate a way to vary the “shadow of the future”. Instead, the model is set up in a way that any cleaner–client pair only meets once (or does not remember past interactions). Cleaner decision rules are thus only driven by the goal to maximize food intake within every single interaction. The results of this simple model will explain why we did not explore the effects of an iterated interaction structure. The complete formal aspects of the model are detailed in the Materials S6.

### Empirical research

Field observations and the experiment were carried out at the Richard B. Gump South Pacific Research Station in Moorea in February/March 2022, based on preliminary observational and experimental data from 2016. Our research study adheres to the ASAB/ABS Guidelines for the Use of Animals in Research. The study was approved by the local authorities responsible of the program “Délégation à la Recherche” and by the Gump Research Station where the study was conducted in accordance with their rules and regulations for animal research (permit 11626/VP/DRM). We acknowledge that catching is a stressful event for the fish. Afterwards, the fishes adapt well to captive conditions and lose their shyness towards human experimenters. The cognitive experiments can be seen as behavioral enrichment, which studies have shown have to be beneficial for the overall welfare of fish in captivity (Gatto et al. 2024; Silvia et al. 2025). Furthermore, this specific cognitive task closely resembles their natural foraging behavior. The experimental paradigm uses Plexiglas plates, prawn, and fish flakes as direct substitutes for live clients, client mucus, and ectoparasites. By requiring the cleaners to make repeated decisions about feeding on preferred versus less-preferred food items, the task actively simulates the natural decision-making and foraging challenges they experience on the reef (Bshary and Grutter 2005). All fish were released at their site of capture.

During field observations, video material of the two study species was collected using GoPro hero 9 or 8 cameras during 49 dives on adult individuals of either sex (which was not determined) on the fringing reef of the Cook's Bay. Filming took place mostly early morning or late afternoon, ie during times when cleaners are known to be more active (Grutter 1996). Interactions were filmed with at a distance of 2 to 3 m. A lead diver would follow a specific cleaner for about 40 min. A dive buddy would film opportunistically other cleaners when encountered. In total, we obtained 33.5 h of footage. We had 66 video recordings for *L. bicolor* and 50 video recordings for *L. dimidiatus*. The aim was to document interactions in which the client jolted at least twice in response to cleaner fish mouth contacts. Past research has shown that such jolts are a correlate of cleaners eating mucus and hence cheating (Bshary and Grutter 2002). To facilitate later video analysis, interactions were observed directly during filming, and any interaction with at least two jolts was flagged by placing a hand briefly in front of the lens directly afterwards.

All other interactions were not considered. The start of an interaction was defined as the first contact between cleaner and client. We extracted the duration from the beginning of the interaction until the first jolt, and the time between the first and the second jolt. The same individuals could be filmed more than once, which had to be noted for the statistical analysis. *L. dimidiatus* individuals were identified through their location, while unique color patterns verified from footage were used to identify *L. bicolor* individuals instead (Table S1). We obtained 25 interactions of interest for 14 *L. bicolor*, and 63 interactions for 18 *L. dimidiatus*.

During the laboratory experiment, a total of 12 individuals of each species were tested during two rounds of experiment. For each round, we collected six adult *L. dimidiatus* (total length (TL): 6 to 9 cm) and six adult *L. bicolor* (TL: 7.6 to 10.5 cm) of unknown sex. Fish were collected from the fringing reef of the Cook's bay using barrier (1.5 × 1 m with 5 mm mesh size) and hand nets (15 × 15 cm with 5 mm mesh size). After each round, fish were released at site of capture.

Fish were kept in a total of 6 plastic aquaria (66 × 50 × 46 cm, 108.9 L) with direct seawater flow and a small pipe tube as shelter. Each tank contained one fish per species separated by an opaque partition (50 × 46 cm) inserted in the middle. The positioning of individuals of the two species was counterbalanced within tanks. During a 10-d period, fish were acclimated to the laboratory conditions and the presence of the experimenter. Fish were fed by scattering mashed prawn on a Plexiglas plate left in each tank compartment all day. The prawn mash was freshly made every morning. As expected, all *L. dimidiatus* quickly learned how to feed off the plate (Bshary and Grutter 2005). In contrast, all but one *L. bicolor* ignored the food. To facilitate learning, we let them first observe *L. dimidiatus* feeding off a plate by replacing the opaque partition with a transparent one, and then we removed the barrier so that both cleaners could access the same plate (MA personal observation). 7 individuals started foraging after such exposure. The final 4 individuals started eating off the plate when a laminated picture of a conspecific was attached to the plate. During the following days, fish were trained to find small discrete items of prawn placed on black dots painted onto plates. Training was repeated multiple times per day allowing fish to get used to a daily participation to many trials. Each day, the experimenter made sure fish ate until satiation, which took 6 to 10 trials.

The experimental paradigm was based on Bshary and Grutter (2005), testing cleaners' willingness to feed against preference in order to prolong an ongoing interaction with a Plexiglas plate as surrogate of a client. The two food types were pure prawn and prawn mixed with fish flakes (further called “flakes”). Preferred food (prawn) would be the equivalent of client mucus, less-preferred food (flakes) the equivalent of ectoparasites (Grutter and Bshary 2003). Eating the latter was always allowed, while eating the former would cause the removal of the plate with a certain probability. The experiment comprised three phases: an initial preference test to ensure the fish preferred prawn to flakes; a training phase in which fish were exposed to the negative consequences of eating prawn; and finally the experimental phase to investigate how willingness to feed against preference, as an indicator of cooperativeness, changes over the course of an interaction.

The preference test was performed on the 11th day after capture. This phase had two goals: (i) to ensure the cleaner showed a preference for prawn, and (ii) following the protocol of Bshary and Grutter (2005) to create for each individual a mixture of prawn and flakes (“flakes”) that would be less preferred than prawn but still be eaten. During this phase, the fish did not experience any adverse consequences related to eating its preferred food item. The details are described in Materials S2.

Before starting the experiment, cleaners needed to learn about the consequences of eating the two food items, ie that prawn consumption causes the removal of the plate while flakes consumption never causes plate removal. Therefore, the 12th and 13th days were used for training. During training, the probability of removal after prawn consumption was 100% to maximize chances that cleaners learn the contingency. An approximately 60 min intertrial interval was kept and each fish underwent between 5 and 8 trials per day. Following methods presented in Gingins et al. (2013), we designed a new Plexiglas plate (13.5 × 10 cm) attached to a 23 cm wooden lever on which 15 flakes (red spots) and 3 prawn (black spots) items were presented within a 6 × 3 grid (Figure S3). The sequence of prawn and flakes was randomized only once and reused with each individual for the training phase and later for the experiment. During a training trial, the plate was introduced to the aquarium three times and removed when the cleaner ate a prawn item. The removal was so fast that no additional item could be eaten. After the first and second prawn item consumption, the plate would be re-introduced into the tank after having been kept out of sight for 60 s without re-filling the eaten items. A trial normally ended with the consumption of the third prawn item. However, if the fish had not eaten any flakes prior to the three cheating opportunities, the plate would be re-introduced once more so that cleaner could eat flakes and potentially learn the lack of consequence linked to foraging on flakes. Individual adjustments on the flake concentration inside the mixture were made in order to make sure that despite disliking it they would still eat some flakes prior to cheating. We aimed for an average of 2 to 3 flake items eaten before a prawn, which worked for most individuals (Materials S2). This specific target was chosen because complete avoidance of prawn would have prevented us from measuring multiple cheating intervals, whereas immediate prawn consumption would end the trial instantly. By ensuring they ate 2 to 3 flakes first, we established a baseline delay that allowed us to test during the actual experiment whether the ratio increase or decrease over the course of a single interaction when removal probability is <100% (see below).

The experiment started on the 14th day and lasted 8 d. We conducted six trials per fish on the first day due to time constraints, and 12 per day thereafter. The plate was rapidly removed after a cheating event with a 50% chance and not reintroduced until the next trial, approximately 60 min later. Therefore, 50% of the trials ended after the first cheating event, 25% after the second and the remaining 25% after the third. Every day a new sequence of responses was randomized and counterbalanced for each individual, to ensure that each fish experienced all three responses. In trials in which cleaners could eat at least two prawn items before the plate was removed, we quantified the number of flake items eaten before the first prawn item, and the number of flakes eaten between the first and second prawn item. Whenever the cleaners could also eat the third prawn item, we also counted the number of flake items between the second and the third prawn. Trials in which the plate was removed after the cleaner ate the first prawn item were discarded. Sometimes, cleaners revisited a spot from which they had already eaten and touched it again with their mouth, potentially because a residual amount of food had remained. We decided not to count trials in which the fish ate twice from the same prawn spot. However, the trial was counted if the fish came back to the same flake spot and two separate tables where created, one in which coming back to a previously visited flake spot was counted as an additional eaten item, and another in which it was not. Since the two tables produced equivalent results (Appendix S5), we decided to use the second one in our final data analyses to remain conservative. Foraging on an already visited prawn spot was not a rare event especially for small sized fish. Therefore, we repeated such failed trials at the end of the day if fish were still hungry and motivated, alternating with a control trial in which the plate was removed after the first cheating event.

### Statistical methods

#### Fieldwork

The two durations (start of interaction until first jolt, duration between first and second jolt) formed matched data pairs. Using R software 4.1.0 (RStudio Team 2020), we fitted a linear mixed effect model (LMER). We computed an analysis of covariance to investigate if (i) the duration (in s) of the first and second intervals was significantly different for both species and (ii) if the trend might be different between species. The natural logarithm of interval duration (ln(duration +0.1)) was used as the response variable, Species and Interval as fixed factors, and the random factor of Fish-id nested in Interaction-id was investigated on the intercept, including a random factor allowed for the data to be recognized as matched by the model.

#### Laboratory experiment

For the initial preference test, two nonparametric Wilcoxon matched-pairs tests were used to evaluate if the two species differed in their preference. This analysis was performed by the mean of the package “stats” (R Core Team 2021). For the experiment, we fitted a first generalized linear mixed effect model (GLMER) following the negative binomial distribution by the mean of the R package “lme4” (Bates et al. 2015) and computed an analysis of covariance (ANCOVA) using the “Anova” function of the “car” package (Fox and Weisberg 2019). First of all, this model aimed to investigate whether the amount of flakes eaten during the first and second intervals differs for both species. Second, we wanted to investigate whether the latter difference is the result of learning rather than something they naturally do. That is, whether or not the number of flakes eaten during the two intervals changes over the course of the experiment. Third, we wanted to explore if there is a different trend between species. The number of flakes consumed was used as a response variable (Items) while Interval (Interval 1 and Interval 2), Species, Time, and their interactions were used as fixed factors, and Fish ID nested in Trial ID was investigated as a random factor on the slope and the intercept. The random factor enabled our model to recognize the data as matched samples. Exploring Time as an explanatory variable enabled us to include all trials from the experimental phase in our analyses without distinguishing between acclimation and experimental trials. A second model, similar to the above-mentioned one, was then used to extend these questions to the third interval using a data set containing only interactions with three cheating events. Following the approach from Oates et al. (2010a) who specified that little is known about the reproductive system and related behavior of *L. bicolor*, we did not use sex as a parameter of interest in our analyses with either model. This decision is also based on the fact that determining the sex of *L. bicolor* is difficult and requires specific manipulations, for which we had not applied in our ethics permit. The same analyses were run with the tables in which coming back to a previously visited flake was counted as an additional eaten item (Materials S5).

All statistics were performed on the R software version 4.1.0 (RStudio Team 2020). Pairwise comparison on the intercepts and slopes were obtained using the R package “emmeans” (Russel 2022).

## Results

### The model

Under the assumptions laid out in Fig. 1, the model predicts a monotone decrease in cleaner fish exploitation rates over the course of an interaction (Fig. 2). As an interaction continues, the cleaner does best to become less exploitative, reflecting the decreasing willingness of clients to put up with the costs imposed on them (due to their decreasing marginal benefits of continued interaction).

**Figure 2:**
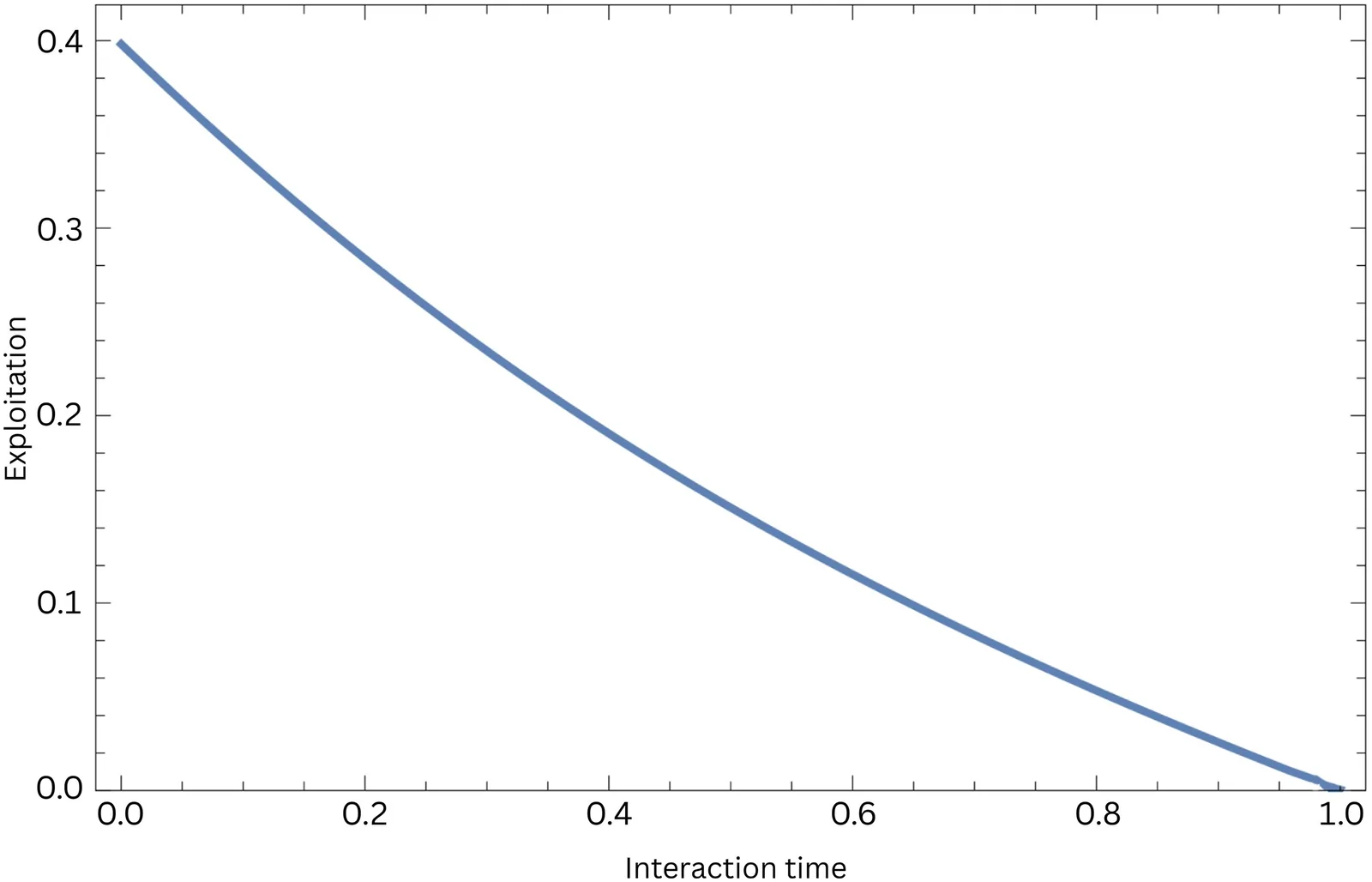
The level with which cleaners exploit clients generally declines as a function of interaction time under the assumption that variation in client needs follows a beta distribution.

Importantly, the cleaners' level of exploitation does not fall as fast as the willingness of clients to tolerate it. As a consequence, as an interaction continues, the client becomes more and more likely to leave (Fig. 3). While all clients would eventually leave anyway, as their marginal benefit dropped to zero (yellow line in Fig. 3), cleaners speed up this process by exploiting the clients. It is worth noting that exploitation by cleaners causes a proportion of interactions to never take place, because clients with little need would experience a negative payoff and hence avoid the interaction. The result can also be viewed in the opposite direction: cleaners do best to “give up” on these low need clients by choosing a level of exploitation they will not tolerate, as such a level allows them to extract more benefits from needier victims.

**Figure 3:**
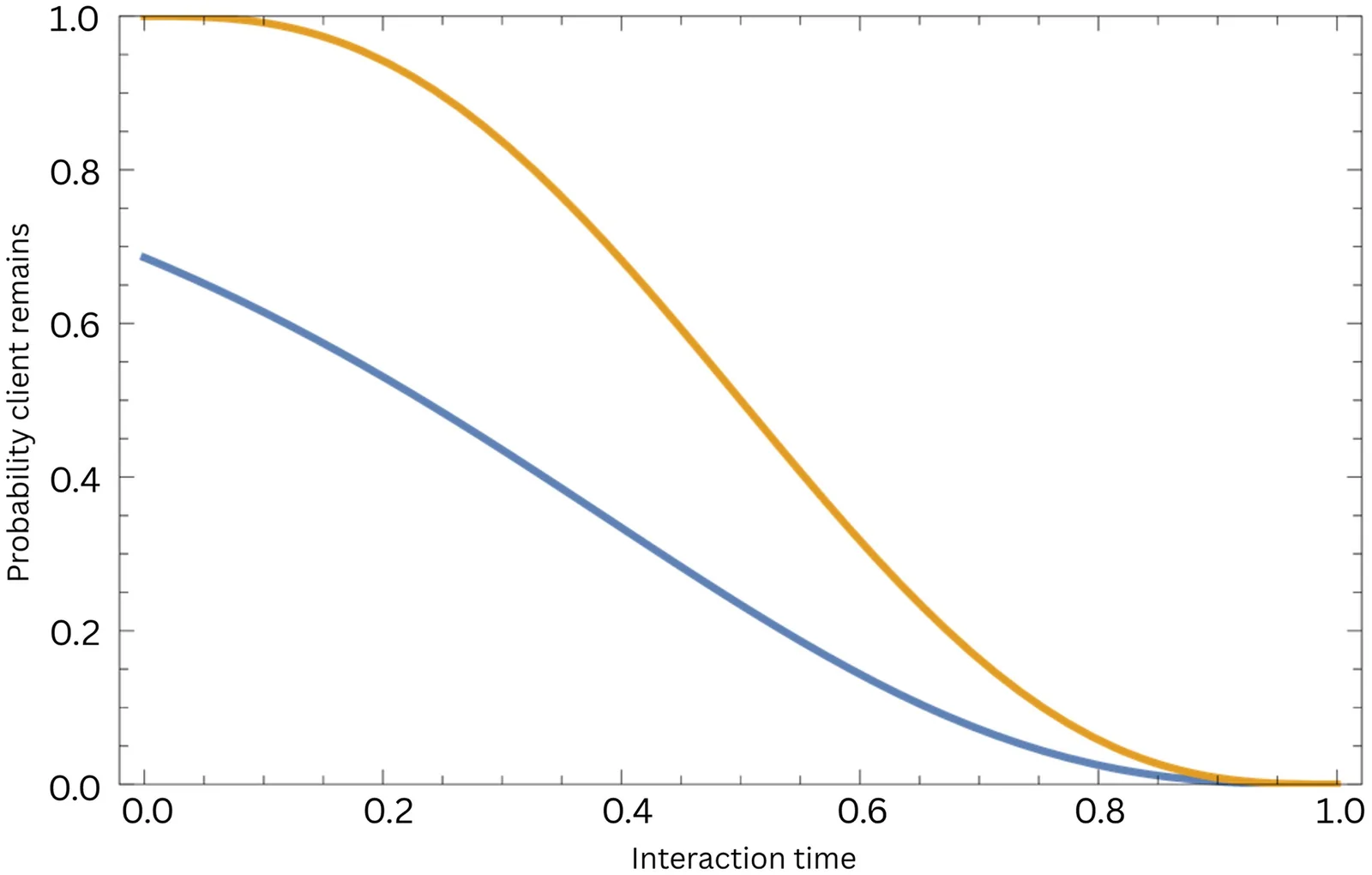
Two functions depicting the percentage of clients remaining as a function of interaction time. The yellow line shows the proportion of clients that would willingly remain if the cleaners were not exploiting at all. The blue line shows the relationship given the cleaners' level of exploitation. Some clients have such low needs for cleaning that their best response is to avoid any interaction because the cleaners' exploitation rate would yield negative payoffs.

Also of interest is that the ratio of the solid to the dotted line in Fig. 3 decreases over time; in other words, of those clients that would willingly remain for any given length of time, the cleaner “gives up” on an increasing fraction as the interaction continues (pushing the client to leave by not reducing its rate of exploitation fast enough). Functionally speaking, the cleaner is getting increasingly “impatient” with low-need clients as time goes on.

Only if we assume that variation in client needs is best described with a log-normal distribution (Fig. 4a), levels of exploitation may eventually increase again after an initial decline (Fig. 4b). However, such events would be extremely rare as most interactions would have been long terminated before cleaners decide to increase their exploitation again (Fig. 4c). A log-normal distribution of client needs apparently causes cleaners to reassess their own estimate of a client's current needs during an interaction, where a very long interaction suggests that the need is higher than anticipated and hence allows for higher exploitation rates. Given the rarity of such events, observers in the field would still mostly document the initial decline in exploitation rates as a function of interaction duration.

**Figure 4:**
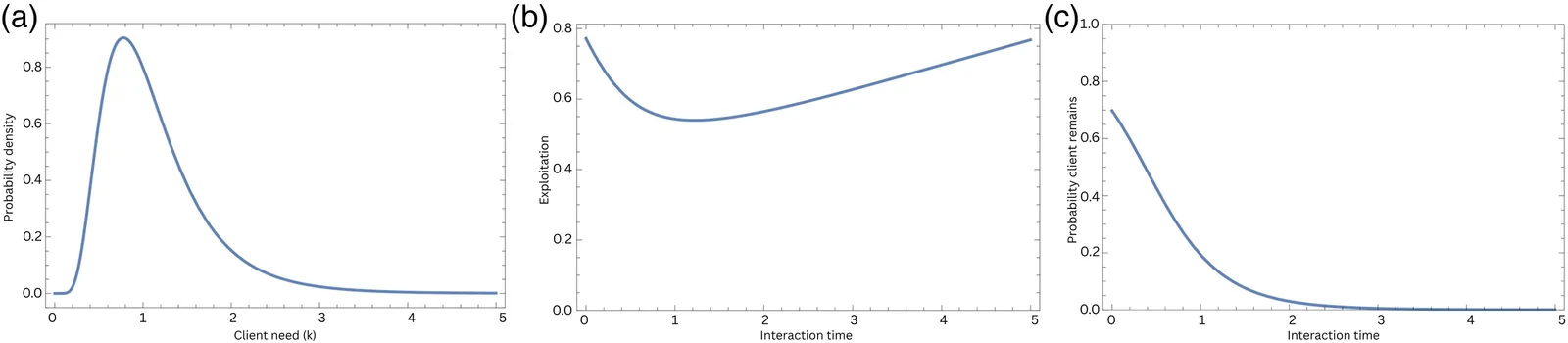
Alternative assumption that client needs are best described by a log-normal distribution a). Under these circumstances, cleaner fish exploitation rate initially decreases but eventually increases again b). Such events would be very rare because the vast majority of interactions would be terminated before this point in time is reached c).

Observations Video recordings yielded 23 interactions of interest for 14 *L. bicolor* (Materials S1 for individual recognition information), and 61 interactions for 18 *L. dimidiatus*. Overall, the second interval (ie, the time between the first and second jolt) lasted significantly longer than the first one (ie, the time between the beginning of the interaction and the first jolt) (Analysis of deviance: Chisq = 15.05, df = 1, *P* < 0.001). This observation holds for both species (Fig. 5) as the effect of interval was not mediated by Species (Analysis of deviance: Chisq = 1.74, df = 1, *P* = 0.18). Post hoc analyses revealed a significant effect of interval for both *L. bicolor* (estimate = −0.634, *t*(82) = −3.154, *P*-value = 0.0022), and *L. dimidiatus* (estimate = −0.323, *t*(82) = −2.616, *P*-value = 0.0106) on the log scale. Back-transformed estimated marginal means indicate that mean duration increased from Interval 1 to Interval 2 by approximately 88.5% for *L. bicolor* and 38.1% for *L. dimidiatus*. Our model explains 38.8% of the variation.

**Figure 5:**
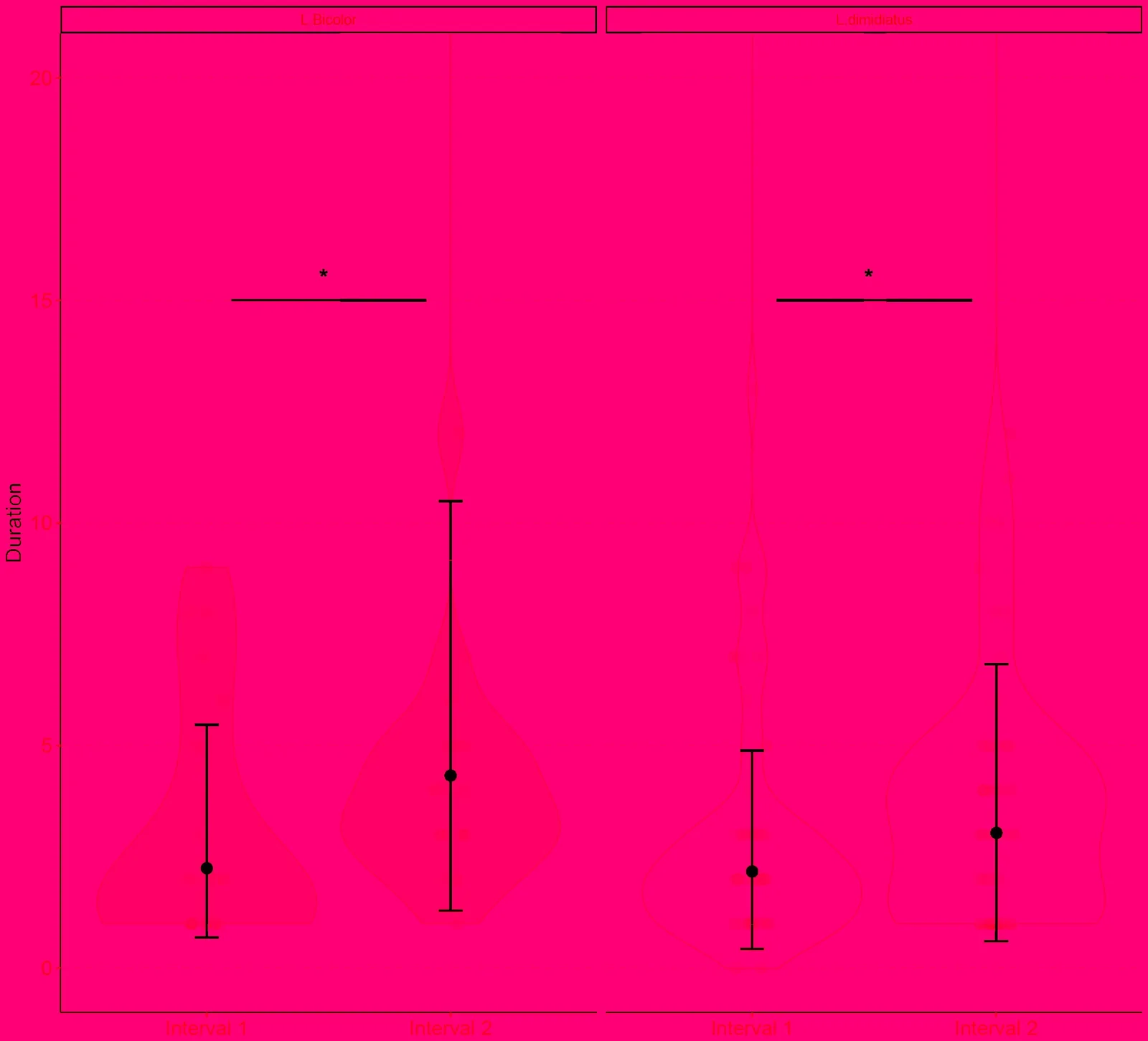
Distribution of interval duration for each video of each Fish ID shown as violins. The jitter represents the observed interval duration between the onset of an interaction and a client's first jolt (Interval 1) and between the first and second jolt (Interval 2). Overlaid points and error bars represent model-estimated means ± standard error (SE). The left plot depicts results for *L. bicolor*, the right plot for *L. dimidiatus*.

### Laboratory experiments

Twenty-three fish passed the preassigned criterion of showing at least 6/7 preference for pawn over three consecutive choice trials. The 24th individual started eating less than 7 items per trial. Increasing the number of trials, prawn was constantly the dominant food type, hence we considered the individual to be suitable for the experiment as well. This preference was further confirmed to be highly significant over all testing trials for both *L. dimidiatus* (Wilcoxon signed rank test with continuity correction: *n* = 12, *V* = 0, *P*-value < 0.01) and *L. bicolor* (Wilcoxon signed rank exact test: *n* = 12, *V* = 0, *P*-value < 0.001). Therefore, 100% of the subjects developed a preference for Prawn and ate more Prawn than Flake items when allowed to forage freely without plate-removal consequences. Summing up all experimental trials, the lowest individual preference was 29 prawns to 13 flakes (64%) for both species. Thus, all individuals could proceed to the training phase and the experiment.

Due to varying satiety levels, we performed in total between 81 to 102 and 88 to 104 trials with *L. dimidiatus* and *L. bicolor* individuals respectively. Of these, 27 to 52 and 34 to 51 trials for *L. dimidiatus* and *L. bicolor* respectively contained at least two prawn consumptions. Two *L. bicolor* did not complete the experiment, either because of a lack of motivation or because of an apparent shift of preference (stopped eating prawn). Each of these two individuals underwent only 55 trials, of which less than 10 could be included in the analyses.

The overall number of flakes consumed during the second interval was significantly higher than the amount consumed during the first interval (Analyses of deviance: Chisq = 147.20, df = 1, *P* < 0.0001; Fig. 6b). Both species ate significantly more flakes during the second interval, compared with the first one as there was no significant interaction between the effect of interval and species (Analyses of deviance: Chisq = 2.94, df = 1, *P* = 0.0866).

**Figure 6:**
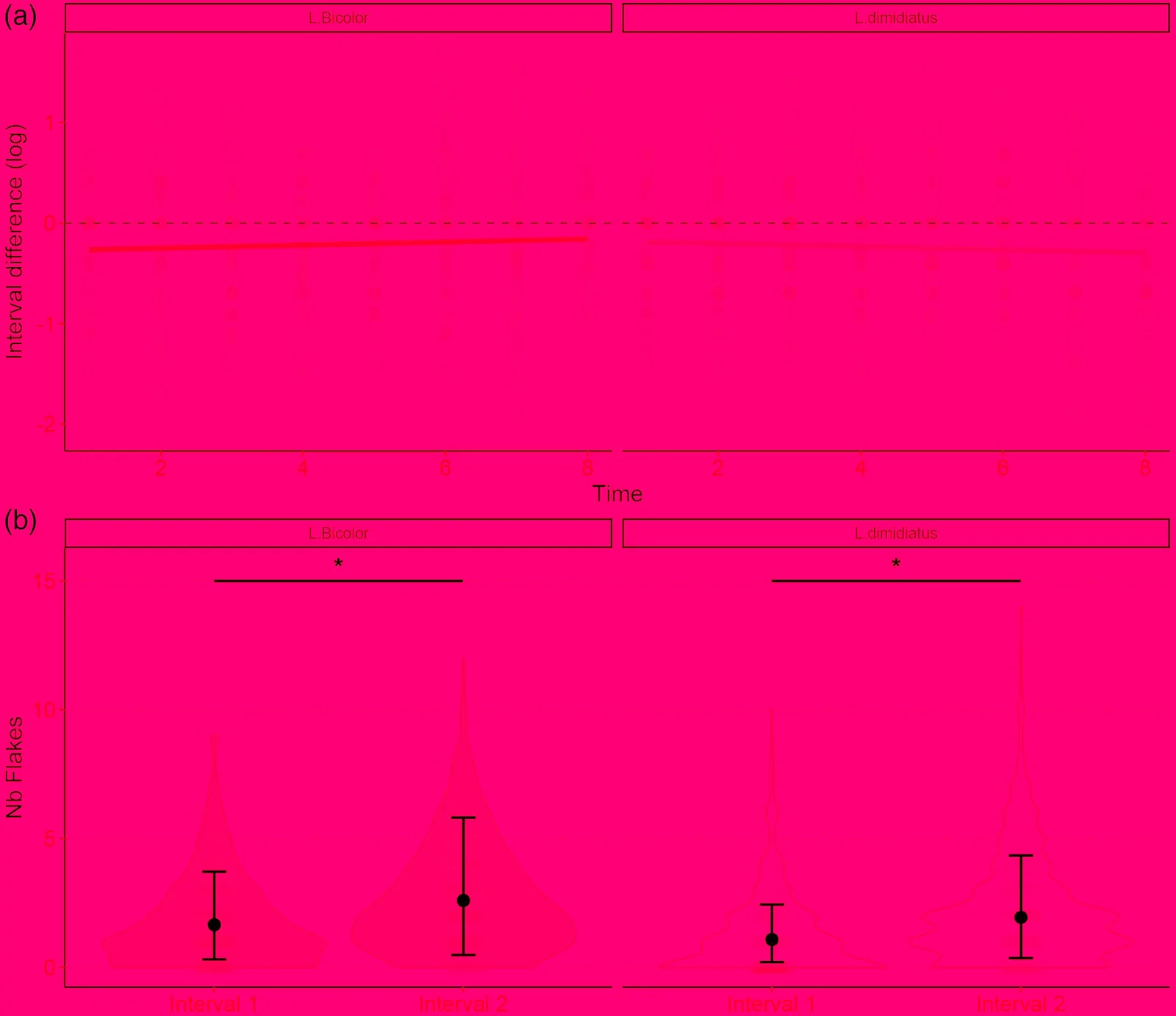
Scatter plot of the difference in the number of flakes eaten during the first and second interval over time (days of experiment) for both species a). Distribution of the number of Flakes items eaten during the first and second intervals shown for each species as violins. The jitter represents the observed number of Flakes, and overlaid points and error bars represent model-estimated means ± standard error (SE) b). In both panels the left plot depicts results for *L. bicolor*, the right plot for *L. dimidiatus*.

The effect of Interval was not affected by Time (trial number) (Analyses of deviance: Chisq = 0.58, df = 1, *P* = 0.45; Fig. 6a). A post-hoc analysis comparing the slopes of the two intervals over time for both species further yielded no evidence for a learning curve by highlighting a lack of significant change over time in the difference between the two intervals for both species (*L. dimidiatus*: *P* = 0.73; *L. bicolor*: *P* = 0.19). This first model resulted explaining 22% of the variation.

When considering all three possible intervals, Interval represented again a significant explanatory variable (Analyses of deviance: Chisq = 113.3, df = 2, *P* < 0.0001) with the effect of Interval differing across Species (Analyses of deviance: Chisq = 6.28, df = 2, *P* = 0.044). Post-hoc analyses confirmed that the number of flakes consumed during the second interval was significantly higher than the number consumed during the first interval for both species (*L. dimidiatus*: *P* < 0.0001; *L. bicolor*: *P* < 0.0001). In contrast, the number of flakes eaten during the third interval was significantly higher than during the second interval only for *L. dimidiatus* (*P* < 0.007) but not for *L. bicolor* (*P* = 0.7944; Fig. 7b). In the overall analysis, the effect of Interval was mediated by Time (Analyses of deviance: Chisq = 15.4, df = 2, *P* < 0.001; Fig. 7a). A post hoc analysis comparing the slopes of the three intervals over time for both species revealed that the latter result is due to a significant change over time in the difference between the second and third intervals for *L. bicolor* (*P* = 0.0162; Fig. 7b). Other pairwise comparisons showed no effect of Time on the difference between intervals (I1–I2, *L. dimidiatus*: *P* = 0.983; I1–I2, *L. bicolor*: *P* = 0.993; I2–I3, *L. dimidiatus*: *P* = 0.279). This second model resulted explaining 18.45% of the variation.

**Figure 7:**
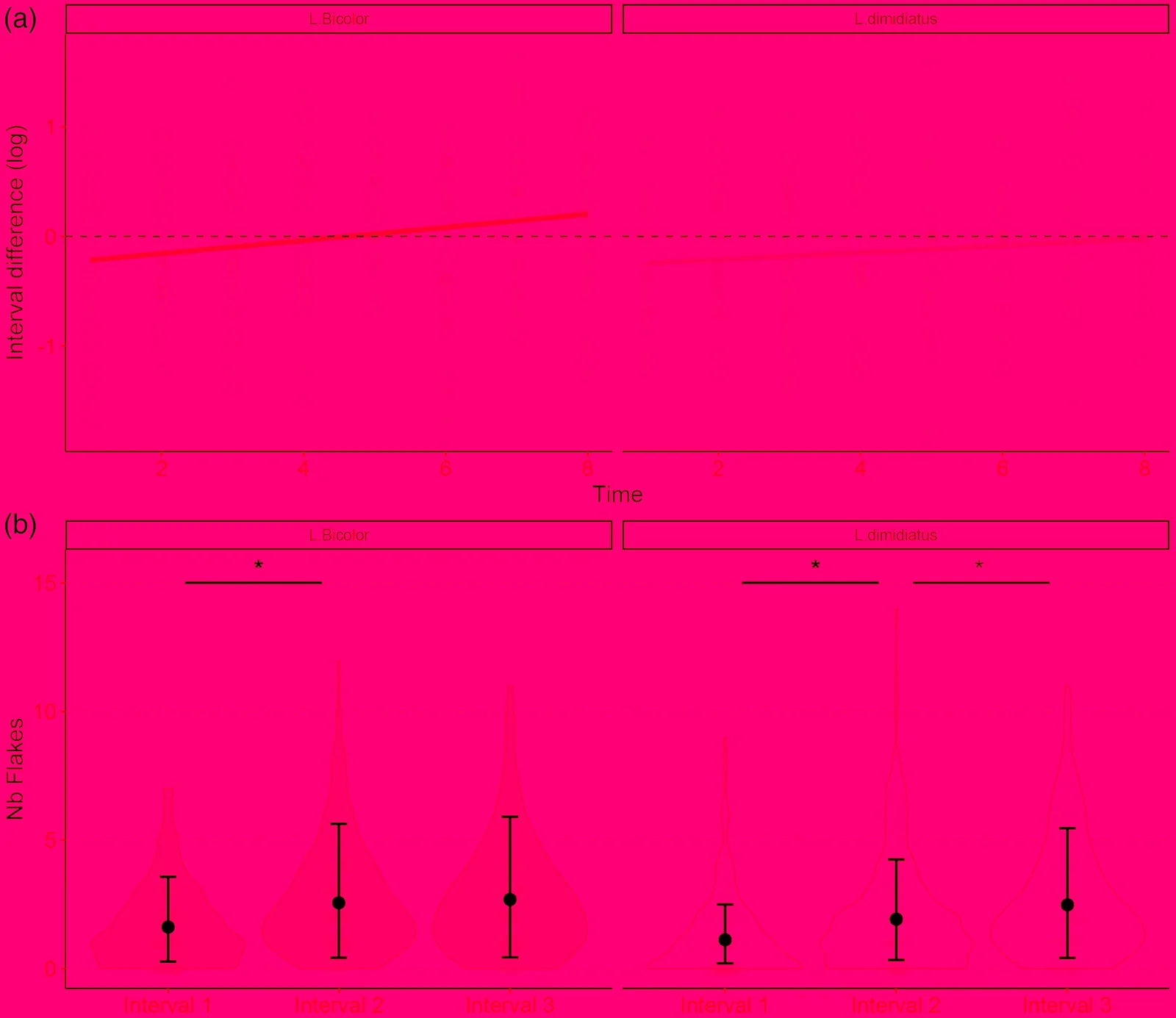
Scatter plot of the difference in the number of flakes eaten during the second and third interval over time (days of experiment) for both species a). Distribution of the number of Flakes items eaten during the first, second and third intervals shown for each species as violins. The jitter represents the observed number of Flakes, and overlaid points and error bars represent model-estimated means ± standard error (SE) b). In both panels the left plot depicts results for *L. bicolor*, the right plot for *L. dimidiatus*.

## Discussion

We had asked how levels of cooperation should change over the course of an interaction between two partners defined by the following features: (i) one partner (the “client’) cannot cheat but can terminate the interaction at any time; (ii) this partner seeks a service but risks being exploited by the other one; (iii) the value of the service decreases with increasing interaction duration. The potential cheater should adjust its service quality (the balance between cooperative and defective acts) accordingly to maximize its own payoffs. Our game-theoretical model showed that under a wide parameter space, such conditions lead to potential cheaters being less exploitative the longer an interaction lasts. Only a highly skewed distribution of client needs would cause service providers to increase exploitation rates at the end of very long-lasting interactions. This eventual increase is best explained by the service provider reconsidering its expectation of how much the client needs the service. The fact that a current client has not terminated an already long-lasting interaction despite the service provider giving an average service indicates that this client is one of the rare cases with a very high need, in which case it would also tolerate a high exploitation rate. We interpret the model's standard prediction as follows: client responsiveness represents an important factor for the potential cheater's decisions about current service quality. The best response to the clients' decision rule is to match own exploitation rates, accordingly, aiming to always stay just below the threshold that would cause clients to terminate the interaction prematurely. This way, the service provider maximizes its chances of benefiting from the cheating acts without experiencing the costs linked to such behavior. Importantly, the model predicts a negative slope in a single interaction; potential consequences on future interactions do not need to be considered. This prediction is independent on the benefits a cleaner gets from each mucus bite, a variable that we have explored in previous models on mean service quality (Johnstone and Bshary 2002, 2008). Further modeling and testing could explore how variation in benefits of cheating (ie client mucus quality) and variation in client needs (ie ectoparasite load) combine to affect payoffs for both cleaners and clients.

We tested the model's prediction on two cleaner fish species. Based on optimal foraging theory (Charnov 1976), one could have predicted cheating rates to increase over the course of an interaction as a consequence of decreasing client quality as a food patch (in terms of ectoparasite load). Considering only client depletion, the temptation of cheating increases over the course of an interaction as the cost of a client potentially terminating an interaction decreases over time (Johnstone and Bshary 2002). On the other hand, an earlier experiment had shown that through interactions, surgeonfish clients adjust the probability of aggressive responses to cheating by cleaners to their own parasite load; highly parasitized clients were less likely to respond aggressively than less parasitized ones, and clients were least tolerant once nearly parasite-free (Bshary and Grutter 2002). Such adjustments of mean tolerance are also predicted in a recent game-theoretical model (Triki et al. 2022). Both field observations and the laboratory experiment clearly fit our model prediction in that cleaners also adjust service quality to shifting client tolerance levels within an interaction. They frequently cheat early on during an interaction and then increase their levels of cooperation. The experimental data confirm that field results were indeed the product of changes in cooperation levels rather than changes in nibbling rates. The analysis, considering data in which cleaners were allowed to cheat three times instead of just two, further confirms that cheating rates do not increase over time. The lack of learning curves during experiments and the similarity of results to field observations suggest that both species seem to be applying their natural decision rules to an abstract version of their interactions. This interpretation fits various other results on *L. dimidiatus* in experiments of ecological relevance (Wismer et al. 2014, 2019; Triki et al. 2020). Thus, the current study adds evidence that experiments using moveable plates, flakes and prawn as substitutes for clients, ectoparasites and mucus capture cleaner fish behavior in nature.

The fact that the same tendency was found with both species despite their ecological differences suggests that the presence or absence of repeated interactions is not a major factor in our particular game. Game theory and iterated prisoner's dilemma-type experiments on humans show the opposite pattern: iteration promotes cooperation but finite horizons (a reduction and eventual complete lack of the “shadow of the future”) promote defection and lead to “end game effects”, ie to a gradual breakdown of cooperation (Milinski et al. 2002; Bó 2005; Normann and Wallace 2012). This raises important questions on why the dynamics appear to differ between our game and standard iterated prisoner's dilemma games. We consider two promising variables that should be introduced in an iterated prisoner's dilemma analysis to investigate whether either or their combined addition precludes or even reverses end effects: the possibility to terminate a game prematurely and diminishing payoffs as the game progresses, where the function for each player is only known to self.

Taking into consideration that the interactions of *L. dimidiatus* typically fit a repeated interactions structure, the last decision made during a single interaction is not the very last one, but decisions continue to be made during future interactions with the same client. This “shadow of the future” could have been an additional factor promoting *L. dimidiatus* individuals to refrain from cheating towards the end of an interaction. In contrast, the interactions of roving adult *L. bicolor* often resemble one-shot interactions as their movements prevent clients from actively choosing or avoiding them, and the unpredictability of encounters may cause both cleaner and client not remembering past interactions. As a consequence, the last behavior of a cleaner during a single interaction often constitutes the effective end of the game with the current client (a new interaction would be treated as a new game). Hence, the logic of the “shadow of the future” would have led to predicting an “end game effect” of increased cheating by *L. bicolor* individuals.

Data indicate that the “shadow of the future” logic applies to mean cooperation levels of cleaner fish: *L. dimidiatus* individuals cheat less frequently than *L. bicolor* individuals do (Mills and Côté 2010; Oates et al. 2012), and it has also been observed that *L. bicolor* individuals are less exploitative in the core areas of their home ranges, where chances of re-interacting with the same client in a short period of time are higher (Oates et al. 2010b). Our model, however, does not investigate mean exploitation levels but instead predicts that exploitation should decrease within a single interaction without any consideration of future interactions. Correspondingly, the two species behaved very similarly, lowering exploitation rates over the course of single interactions in both field observations and laboratory experimental results.

In conclusion, our study emphasizes the importance of changing needs (payoffs) over the course of interactions for prediction changes in cooperation/exploitation. Changes in needs over the course of interactions often occur in natural systems. Many mutualisms involve food or nutrients as a resource (Bronstein 1994), where uptake yields a benefit function of diminishing returns. In many such cases, food or nutrient providers are plants or microbes that lack the possibility to respond in real time to the partner's behavior. It would hence be interesting to study such systems in more detail. Cooperative hunting and group territory defense provide opportunities to study changes in individual contributions within an event in *N*–player interactions. For cleaning mutualism, a more detailed future study could evaluate whether cleaner fish adjust service quality over the course of an interaction as a function of client species. Different traits are known to influence mean service quality, such as mucus load, ectoparasite load and home range (Roche et al. 2021), and client selectivity and tolerance are predicted to affect mean service quality as well (Johnstone and Bshary 2008; Triki et al. 2022) but whether specific trait combinations lead to different slopes of exploitation over the course of single interaction is an open question. Furthermore, the proximate mechanisms leading to increased levels of cooperation over the course of an interaction need investigation. Shifts in the internal state of cleaners due to partial satiation on the preferred item appear to be unlikely to explain the results as individuals readily consumed multiple prawn items in a row during the initial preference test, when no negative consequences were applied. Instead, this shift likely reflects a learned adjustment of perceived risk of the client leaving. Finally, our results show that “end game effects” are not inevitable when partners have at least increased expectations of an interaction ending. Theoretical research could investigate in more detail whether variables that counter the effect of finite horizons in our game have similar effects in a variety of repeated games.

## Supplementary Material

arag121_Supplementary_Data

## Data Availability

The analyses reported in this article can be reproduced using the data provided by Pessina et al. (2026).
