# Supplementary material for: Levels of cooperation increase during interactions with iterated decision-making
Source: Behav Ecol. 2026 Sep 30;37(6):arag121. doi: 10.1093/beheco/arag121 (PMC13684399; doi:10.1093/beheco/arag121)
Supplement: Supplementary file 1 [file arag121_supplementary_data.docx]

# **Supplementary Materials**

Supplementary tables and information related to the manuscript titled **“Levels of cooperation increase during interactions with iterated decision-making: a model and case studies on two cleaner fish species”.**

[Supplementary Materials	1](#_Toc222209641)

[**Supplement 1: L. bicolor individual recognition**	2](#_Toc222209642)

[Figure S1: Map showing the locations of the 14 *L. bicolor* for which we have double cheating events on camera.	2](#_Toc222209643)

[Table S1: A table of all *L. bicolor* for which we obtained interactions of interest. Individual recognition was performed based on colour patterns	2](#_Toc222209644)

[**Supplement 2: Initial Preference Test**	5](#_Toc222209645)

[Figure S2: The Plexiglas plate for the preference test with the used sequence of Prawn (P) and Flake (F) items.	5](#_Toc222209646)

[Table S2: Preference test results for L. dimidiatus.	6](#_Toc222209647)

[Table S3: Preference test results for L. bicolor.	6](#_Toc222209648)

[**Supplement 3: Training phase.**	7](#_Toc222209649)

[Figure S3: The Experimental Plate.	7](#_Toc222209650)

[**Supplement 4: Flake proportions.**	8](#_Toc222209651)

[Table S4: Calculations of expected average number of flakes eaten before the first prawn and temptation of eating the first prawn after eating the expected amount of flakes.	9](#_Toc222209652)

[Table S5: Calculations of expected average number of flakes eaten before the second prawn and temptation of eating the second prawn after eating the expected amount of flakes.	9](#_Toc222209653)

[Table S6: Calculations of expected average number of flakes eaten before the third prawn and temptation of eating the third prawn after eating the expected amount of flakes.	10](#_Toc222209654)

[**Supplement 5: Analyses where coming back to a previously visited flake counted as an additional eaten item.**	11](#_Toc222209655)

[Figure S4: Difference in flakes eaten before first and second interval.	11](#_Toc222209656)

[Figure S5: Difference in flakes eaten before first, second and third interval.	13](#_Toc222209657)

[**Supplement 6: The model.**	14](#_Toc222209658)

## **Supplement 1: L. bicolor individual recognition**

## **Figure S1: Map showing the locations of the 14 *L. bicolor* for which we have double cheating events on camera.**

## **Table S1: A table of all *L. bicolor* for which we obtained interactions of interest. Individual recognition was performed based on colour patterns**

| Cleaner ID | Left side | Right side | Depth [m] |
| --- | --- | --- | --- |
| Zone 1 | | | |
| Bico_43 |  |  | Average: 5,1  Max: 13,8 |
| Zone 2 | | | |
| Bico_9 |  |  | Average: 3  Max: 5 |
| Bico_17 |  |  | Average: 5  Max: 7 |
| Bico_49 |  |  | Average: 4  Max: 7.3 |
| Zone 3 | | | |
| Bico_2 |  |  | Average: 3.7  Max: 10.5 |
| Bico_3 |  |  | Average: 8  Max: 10.5 |
| Bico_61 |  |  | Average: 5.8  Max: 11.1 |
| Bico_35 |  |  | Average: 2.3  Max: 3.7 |
| Bico_56 |  |  | Average: 5.2  Max: 7.7 |
| Zone 4 | | | |
| Bico_6 |  |  | Average: 6  Max: 9 |
| Bico_25 |  |  | Average: 5.2  Max: 9.1 |
| Zone 5 | | | |
| Bico_21 |  |  | Average: 10  Max:14 |
| Bico_33 |  |  | Average: 4.2  Max: 7.2 |
| Bico_34 |  |  | Average: 5.2  Max: 8.8 |

## **Supplement 2: Initial Preference Test**

The preference test was performed during the 11th day after capture and aimed at creating for each individual a mixture of prawn and flakes (“flakes”) that could be less preferred than prawn but still eaten. We designed a Plexiglas plate (10x7cm) attached to a 23cm wooden lever on which 7 items of each food type were presented within a 5x3 grid with an empty space in the middle (Fig. ESM2). Prawns were put onto black spots and flakes onto red spots. To further help discriminate between the two food types, we added red food colouring to flakes. We randomised and balanced the sequence of prawn and flakes items only once and reused it for each individual throughout the entire process.

## **Figure S2: The Plexiglas plate for the preference test with the used sequence of Prawn (P) and Flake (F) items.**

After two trials to acclimate fish to the unfamiliar plate, we started the preference test. During each trial, the plate was left inside the tank compartment until the fish stopped foraging. We recorded the first 7 eaten items, allowing the fish to eat either food type between 0-100%. We started with a mixture (“flakes”) containing 20% flakes for all fish but made individual adjustments throughout the day. This led to a mixture containing 10-20% and 20-50% of flakes for *L. dimidiatus* and *L. bicolor,* respectively. Fish were tested until a preference of at least 6/7 for prawn was reached for three consecutive trials. Such goal was achieved between 3-7 trials. We maintained an intertrial interval of approximately 60 minutes. Once the preference was determined, fish were normally fed for the rest of the day.

**Table S2: Preference test results for L. dimidiatus.** Percentages of prawn in the first 7 items of food eaten by each *L. dimidiatus* and trial. In green are highlighted the three consecutive trials for which a percentage higher than 6/7 was achieved. The last row specifies final flake percentages (F%) in the mixture.

| % OF PRAWN ITEMS | | | | | | | | | | | | |
| --- | --- | --- | --- | --- | --- | --- | --- | --- | --- | --- | --- | --- |
| **TR** | **D1** | **D2** | **D3** | **D4** | **D5** | **D6** | **D7** | **D8** | **D9** | **D10** | **D11** | **D12** |
| 1 | 71,4 | 42,8 | 57,1 | 71,4 | 85,7 | 42,8 | 71,4 | 100 | 57,1 | 57,1 | 71,4 | 100 |
| 2 | 42,8 | 71,4 | 57,1 | 80 | 57,1 | 100 | 57,1 | 71,4 | 71,4 | 57,1 | 71,4 | 100 |
| 3 | 71.4 | 28,6 | 57,1 | 100 | 85,7 | 100 | 71,4 | 85,7 | 71,4 | 85,7 | 85,7 | 85,7 |
| 4 | 71,4 | 57,1 | 71,4 | 100 | 100 | 100 | 57,1 | 57,1 | 57,1 | 71,4 | 85,7 | 100 |
| 5 | 100 | 57,1 | 100 | 100 | 71,4 | 100 | 71,4 | 100 | 100 | 100 | 100 | 100 |
| 6 | 100 | 85,7 | 85,7 | 100 | 85,7 | 85,7 | 100 | 100 | 100 | 100 |  |  |
| 7 | 100 | 85,7 | 85,7 | 100 | 85,7 |  | 100 | 100 | 100 | 100 |  |  |
| 8 |  | 100 |  | 100 | 85,7 |  | 85,7 |  |  |  |  |  |
| 9 |  |  |  | 100 | 85,7 |  |  |  |  |  |  |  |
| F% | 20 | 20 | 20 | 20 | 15 | 10 | 50 | 20 | 20 | 20 | 10 | 10 |

**Table S3: Preference test results for L. bicolor.** Percentages of prawn in the first 7 items of food eaten by each *L. bicolor* and trial. In green are highlighted the three consecutive trials for which a percentage higher than 6/7 was achieved. The last row specifies final flake percentages (F%) in the mixture.

| % OF PRAWN ITEMS | | | | | | | | | | | | |
| --- | --- | --- | --- | --- | --- | --- | --- | --- | --- | --- | --- | --- |
| **TR** | **B1** | **B2** | **B3** | **B4** | **B5** | **B6** | **B7** | **B8** | **B9** | **B10** | **B11** | **B12** |
| 1 | 57,1 | 28,6 | 42,8 | 42,8 | 57,1 | 42,8 | 85,7 | 71,4 | 71,4 | 57,1 | 100 | 42,8 |
| 2 | 57,1 | 28,6 | 57,1 | 42,8 | 57,1 | 57,1 | 85,7 | 57,1 | 71,4 | 57,1 | 85,7 | 57,1 |
| 3 | 57,1 | 42,8 | 71,4 | 57,1 | 42,8 | 85,7 | 57,1 | 42,8 | 42,8 | 85,7 | 85,7 | 71,4 |
| 4 | 71,4 | 57,1 | 42,8 | 42,8 | 100 | 100 | 100 | 57,1 | 85,7 | 85,7 | 100 | 85,7 |
| 5 | 57,1 | 71,4 | 42,8 | 71,4 | 85,7 | 100 | 71,4 | 57,1 | 71,4 | 100 |  | 100 |
| 6 | 100 | 71,4 | 85,7 | 71,4 | 100 | 100 | 100 | 85,7 | 71,4 |  |  | 100 |
| 7 | 100 | 85,7 | 85,7 | 85,7 | - | - | 100 | 100 | 85,7 |  |  |  |
| 8 | - | 100 | - | 100 | - | - | 100 | 85,7 | 85,7 |  |  |  |
| 9 | 100 | 85,7 | 85,7 | 100 | - | - |  |  | 100 |  |  |  |
| F% | 50 | 50 | 50 | 50 | 30 | 30 | 50 | 50 | 50 | 20 | 20 | 50 |

## **Supplement 3: Training phase.**

During this training phase eating a prawn item would lead to the removal of the plate for 60 seconds.

**Figure S3: The Experimental Plate.** The experimental plate (13.5x10cm) attached to a wooden lever (23cm) with the used sequence of prawn (P) and flakes (F). The colour of the illustrated grey bands on the plate was different for each phase.

## **Supplement 4: Flake proportions.**

Based on the chosen consequences linked to a cheating event, we decided to offer only three prawn items on the experimental plate. We then had to design an experimental plate that offered an appropriate number of flake items relative to the three prawn items. We needed enough flakes so that the cleaner fish could forage naturally and choose to cheat at prawns without being constrained by scarcity. At the same time, we wanted to avoid using an excessively large plate, which would be difficult to handle experimentally and could risk the fish overfeeding, reducing their motivation to continue participating.

To determine if 15 was an appropriate number of flakes, we calculated the expected number of flakes eaten before each of the three prawns under the assumption that fish forage randomly based on availability. For the first prawn, we computed the probability that the fish would eat 1, 2, … up to 15 flakes before encountering the prawn. These probabilities were calculated by considering the order of items eaten; for example, the probability of eating exactly 1 flake before the prawn is the probability that the first item eaten is a flake and the second is a prawn:

$$\frac{15}{18}\times\frac{3}{17}\approx0.147$$

Similarly, the probability of eating exactly 2 flakes before the first prawn is the probability that the first two items are flakes and the third is a prawn:

$$\frac{15}{18}\times\frac{14}{17}\times\frac{3}{16}\approx0.129$$

Multiplying each number of flakes by its probability and summing these products gave the expected number of flakes eaten before the first prawn (Table ESM 4).

For the second and third prawns, the same procedure was applied after removing the expected number of flakes eaten before the first prawn (approximated to 4) and previously eaten prawn, so that the calculations reflected the remaining items on the plate (Tables ESM 5–6).

We also calculated the temptation to cheat at each prawn, defined as the ratio of available prawns to remaining flakes at that moment. This measure reflects the relative likelihood of choosing prawns given availability. 15 Flakes provided sufficient flakes for natural foraging behaviour, maintained comparable temptation across prawns, and kept the plate manageable for the experiment.

## **Table S4: Calculations of expected average number of flakes eaten before the first prawn and temptation of eating the first prawn after eating the expected amount of flakes.**

| Average number of flake items eaten before the first prawn | | |
| --- | --- | --- |
| Nb of flakes | Probability | Nb flakes x Probability |
| 1  2  3  4  5  6  7  8  9  10  11  12  13  14  15 | 0,147  0,129  0,112  0,096  0,081  0,067  0,055  0,044  0,034  0,026  0,018  0,012  0,007  0,004  0,001 | 0,147  0,257  0,335  0,382  0,404  0,404  0,386  0,353  0,309  0,257  0,202  0,147  0,096  0,051  0,018 |
|  | | |
| Average  Temptation at cheating | | 3,748 |
|  |  | 0,266 |

## **Table S5: Calculations of expected average number of flakes eaten before the second prawn and temptation of eating the second prawn after eating the expected amount of flakes.**

| Average nb. of flake items eaten before the second prawn | | |
| --- | --- | --- |
| Nb of flakes | Probability | Nb flakes x Probability |
| 1  2  3  4  5  6  7  8  9  10  11 | 0,141  0,128  0,115  0,103  0,090  0,077  0,064  0,051  0,038  0,026  0,013 | 0,141  0,256  0,346  0,410  0,449  0,462  0,449  0,410  0,346  0,256  0,141 |
|  | | |
| Average  Temptation at cheating | | 3,67 |
|  |  | 0,273 |

## **Table S6: Calculations of expected average number of flakes eaten before the third prawn and temptation of eating the third prawn after eating the expected amount of flakes.**

| Average nb. of flake items eaten before the third prawn | | |
| --- | --- | --- |
| Nb of flakes | Probability | Nb flakes x Probability |
| 1  2  3  4  5  6  7 | 0,125  0,125  0,125  0,125  0,125  0,125  0,125 | 0,111  0,222  0,333  0,444  0,556  0,667  0,778 |
|  | | |
| Average  Temptation at cheating | | 3,11 |
|  |  | 0,257 |

## **Supplement 5: Analyses where coming back to a previously visited flake counted as an additional eaten item.**

This analysis yields the same result as what is presented in the main text (revisits omitted).

The overall number of flakes consumed during the second interval was significantly higher than the number consumed during the first interval (Analyses of deviance: Chisq=153.96, df=1, p<0,001). There was no significant interaction between the effect of interval and species (Analyses of deviance: Chisq=3.8, df=1, p= 0.051), and a post-hoc analysis confirmed that both species ate significantly more flakes during the second interval, compared to the first one *(L. dimidiatus*: p<0.001; *L. bicolor*: p<0.001) (Figure ESM4b). The effect of Interval was not dependent on Time (Analyses of deviance: Chisq=0.462, df=1, p=0.497) (Figure ESM4a). A post-hoc analysis comparing the slopes of the two intervals over time for both species further confirmed the ruling out of a learning curve by highlighting a lack of significant change over time in the difference between the two intervals for both species (*L. dimidiatus*: p=0.749; *L. bicolor*: p=0.238). This first model explained 19% of the variation.

**Figure S4: Difference in flakes eaten before first and second interval.** Scatter plot of the difference in the number of flakes eaten during the first and second interval over time (days of experiment) for both species (a). Distribution of the number of Flakes items eaten during the first and second intervals shown for each species as violins. The jitter represents the observed number of Flakes, and overlaid points and error bars represent model-estimated means ± standard error (SE) (b). In both panels the left plot depicts results for *L. bicolor*, the right plot for *L. dimidiatus*.

When considering the three intervals, Interval represented a significant explanatory variable (Analyses of deviance: Chisq=123.78, df=2, p<0,001), with a marginally significant interaction between the effect of interval and species (Analyses of deviance: Chisq=5.333, df=2, p=0.069). More specifically with a post-hoc analysis the amount of flakes consumed during the second interval was significantly higher than the amount consumed during the first interval for both species *(L. dimidiatus*: p<0.001; *L. bicolor*: p<0.001) while the amount of flakes eaten during the third interval was significantly higher than the one during the second interval only for *L. dimidiatus* *(L. dimidiatus*: p=0.00241; *L. bicolor*: p=0.3145) (Figure ESM 5b). However, the effect of Interval was mediated by Time (Analyses of deviance: Chisq=16.0284, df=2, p<0.001). A post hoc analysis comparing the slopes of the three intervals over time for both species revealed that the latter result was due to a marginally significant change over time in the difference between the second and the third interval for *L. bicolor* (p=0.01) (Figure ESM 5a). Other pairwise comparisons showed no effect of Time on the difference between intervals (I1- I2, *L. dimidiatus*: p=0.999; I1- I2, *L. bicolor*: p=0.996; I2- I3, *L. dimidiatus*: p=0.370). This second model explained 19% of the variation.

**Figure S5: Difference in flakes eaten before first, second and third interval.** Scatter plot of the difference in the number of flakes eaten during the second and third interval over time (days of experiment) for both species (a). Distribution of the number of Flakes items eaten during the second and third intervals shown for each species as violins. The jitter represents the observed number of Flakes, and overlaid points and error bars represent model-estimated means ± standard error (SE) (b). In both panels the left plot depicts results for *L. bicolor*, the right plot for *L. dimidiatus.*

|  |  |
| --- | --- |
|  |  |

## **Supplement 6: The model.**
